# Sub-chronic exposure to second hand smoke induces airspace leukocyte infiltration and decreased lung elastance

**DOI:** 10.3389/fphys.2012.00300

**Published:** 2012-07-27

**Authors:** John M. Hartney, HongWei Chu, Roberta Pelanda, Raul M. Torres

**Affiliations:** ^1^Integrated Department of Immunology, National Jewish Health and University of Colorado DenverDenver, CO, USA; ^2^Department of Medicine, National Jewish Health and University of Colorado DenverDenver, CO, USA

**Keywords:** second hand smoke, inflammation, lung mechanics

## Abstract

Exposure to second hand tobacco smoke is associated with the development and/or exacerbation of several different pulmonary diseases in humans. To better understand the possible effects of second hand smoke exposure in humans, we sub-chronically (4 weeks) exposed mice to a mixture of mainstream and sidestream tobacco smoke at concentrations similar to second hand smoke exposure in humans. The inflammatory response to smoke exposures was assessed at the end of this time by enumeration of pulmonary leukocyte infiltration together with measurements of lung elastance and pathology. This response was measured in both healthy wild type (C57BL/6) mice as well as mouse mutants deficient in the expression of Arhgef1 (*Arhgef1*^−/−^) that display constitutive pulmonary inflammation and decreased lung elastance reminiscent of emphysema. The results from this study show that sub-chronic second hand smoke exposure leads to significantly increased numbers of airspace leukocytes in both healthy and mutant animals. While sub-chronic cigarette smoke exposure is not sufficient to induce changes in lung architecture as measured by mean linear intercept, both groups exhibit a significant decrease in lung elastance. Together these data demonstrate that even sub-chronic exposure to second hand smoke is sufficient to induce pulmonary inflammation and decrease lung elastance in both healthy and diseased animals and in the absence of tissue destruction.

## Introduction

Second hand smoke exposure has been associated with a variety of negative health outcomes in a number of epidemiological studies (Centers for Disease Control, 1986; Barnoya and Glantz, 2005; Eisner et al., 2005; Oberg et al., 2011). As association does not prove causation, animal models are often used to determine causational effects. Accordingly, rodent models of tobacco smoke exposure have been used to establish a relationship between associations identified in epidemiological studies. Historically, these types of tobacco smoke exposures have focused on inducing pulmonary pathology such as tissue damage as indicated by airspace enlargement. The findings from these studies have indicated that relatively long term (>4 months) continual daily exposure to tobacco smoke is required to induce pathological changes in otherwise healthy mice (Hautamaki et al., 1997; Guerassimov et al., 2004; Foronjy et al., 2005; Ma et al., 2005). Furthermore, these types of studies have often used concentrations of tobacco smoke similar to what would be experienced by primary cigarette smokers. Less well studied in murine models is the effect of tobacco smoke at concentrations and for periods of time which reflect second hand smoke exposures in human subjects.

In this study we sought to define the consequences to the murine pulmonary compartment of sub-chronic (4 week) exposure to a mixture of mainstream and sidestream tobacco smoke. We evaluated this exposure in both wild type (C57BL/6) and *Arhgef1*^−/−^ mice. Arhgef1 is an intracellular signaling molecule predominantly expressed by leukocytes and that has been shown to contribute to both leukocyte integrin adhesion and migration (Girkontaite et al., 2001; Rubtsov et al., 2005; Francis et al., 2006; Hu et al., 2008). We have reported that Arhgef1-deficient mice spontaneously develop pulmonary features reminiscent of individuals with chronic obstructive pulmonary disease (COPD; Hartney et al., 2010). These pulmonary features of *Arhgef1*^−/−^ animals include chronic inflammation as defined by elevated numbers of pulmonary leukocytes in lung tissue and the airspace compartment, airspace enlargement and loss of elastic recoil in the pulmonary compartment. More recently, we have identified a novel signaling pathway used by pulmonary macrophages to promote inflammation and that is regulated by Arhgef1 (Hartney et al., 2011). In this study we compare cigarette smoke-induced inflammation in wild type and Arhgef1-deficient animals to determine the relationship between the pathways involved in cigarette smoke exposure inflammatory responses and those dependent on the presence of Arhgef1.

## Materials and methods

### Smoke exposure protocol

C57BL/6 mice were initially obtained from the Jackson Laboratory, Bar Harbor, ME and subsequently bred in our animal facility. Arhgef1-deficient mice were generated and used on a C57BL/6 genetic background (Rubtsov et al., 2005) and also bred and maintained in our animal colony. All experiments with animals were approved by the Institutional Animal Care and Use Committee. Mice were exposed to cigarette smoke (2RF4 reference cigarettes, University of Kentucky) in TE-10z smoking chambers (Teague Enterprises, Davis, CA) for 6 h per day, 5 days per week (Teague et al., 1994). Smoke exposure is adjusted in this system to generate a mixture of sidestream smoke (89%) and mainstream smoke (11%) by burning five cigarettes simultaneously. Chamber atmosphere was monitored for total suspended particulates and carbon monoxide, with concentrations of 70–80 mg/m^3^ and 190 ppm, respectively as previously described (Martin et al., 2006; Tollefson et al., 2010). Animals were sacrificed 1 day after the last smoke exposure and lungs harvested. The 4 week smoke exposure protocol was performed in two separate experiments and cohorts of animals were assessed by leukocyte quantitation, histological examination and lung mechanics on both occasions.

### Enumeration of leukocytes

Lungs were lavaged with Hanks' balanced salt solution with 5 mmol/L EDTA. An aliquot of cells were counted on a Z2 particle count and size analyzer (Beckman Coulter, Fullerton, CA) as previously described (Hartney et al., 2010). Leukocytes were isolated from lavaged lung tissue after treatment with collagenase types II and IV (Sigma-Adrich, St. Louis MO) and dispase II (Roche, Basel, Switzerland), as previously described (Hartney et al., 2010).

### Flow cytometry

After isolation, cells were stained using standard methods and the following antibodies as previously described (Hartney et al., 2010). Leukocytes were identified using a pan-CD45 antibody (30-F11; eBiosciences, San Diego, CA) and F4/80 (A3-1, Serotec, Raleigh, NC), Gr-1 (RB6-8C5; eBiosciences), B220 (RA3/6B2), or CD3 (2C11) for identification of macrophages, neutrophils, B cells and T cells respectively, and back-gated during analysis to confirm appropriate forward and 90° light scatter. CD4 (GK1.5) and CD8 (53-6.7) staining further differentiated T cell subsets. Data were collected with a FACSCaliber (BD Pharmingen, San Diego, CA) and analyzed with FlowJO 8.8.4 software (Tree Star, Inc., Ashland, OR).

### Lung mechanics

Lung mechanics were assessed as previously described (Lovgren et al., 2006) using a Flexivent (Scireq, Montreal, Canada) small animal ventilator. A stepwise inflation up to 1.2 ml of air was applied to the lungs. Pressure-volume graphs were generated with the expiratory phase using the pressure and volume values obtained after a one-second pause in piston movement.

### Lung histology

**Figure 1:**
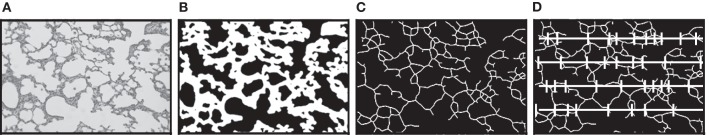
**Schema for the process of quantitating mean linear intercept. (A)** Hematoxylin and eosin stained lung tissue section as an example of the digital images obtained for analysis. **(B)** A binary image of the photomicrograph converted by a macro program. **(C)** The binary alveolar structure is next reduced to a skeletonized structure one pixel in width. **(D)** A series of probe lines are superimposed on the skeletonized image and points where the alveolar structure intercepts the probes are recorded and exported. Mean linear intercept equal total probe length divided by number of intercepts.

Lungs were inflated via tracheal cannula to 25 cm of pressure using a tower filled with 4% paraformaldehyde. The trachea was then tied off below the cannula and the lungs removed and immersed in 4% paraformaldehyde for 24 h. Lungs were then imbedded in paraffin and cut into 2–3 μm-thick slices at a random orientation and stained with hematoxylin and eosin. At least twenty-five 20× fields were captured electronically by stratified random sampling as previously described (Subramaniam et al., 2007). Next we used a digital image analysis approach as described by Tschanz and Burri (2002) and subsequently adapted into a macro for ImagePro 4.5 (Media, Cybernetics; Hartney et al., 2010). This process occurs in three automated steps as illustrated in Figure 1. First the digital image shown in Figure 1A is converted into a binary image (Figure 1B). Next this binary image is skeletonized (Figure 1C). Then a series of probes are superimposed across the skeletonized image (Figure 1D). Points where the skeletonized image intercepts a probe are identified and tallied. The number of intercepts and the length of the probes applied were then reported to a spreadsheet. Mean linear intercept was calculated by the formula:

$$\text{Mean linear intercept}=\frac{\text{Total probe length}}{\text{Total number of intercepts}}$$

### Statistical analysis

JMP® (SAS Institute Inc., Cary NC) was used for all statistical analysis. For data sets including four groups a One-Way ANOVA was performed. If significance was detected with the One-Way ANOVA, a *post hoc* Tukey–Kramer HSD *t* test was performed. To determine whether there was a significant interaction between genotype (B6 vs. *Arhgef1*^−/−^) and exposure [filtered air (FA) vs. second hand smoke (SHS)] a Two-Way ANOVA was performed on all appropriate data sets.

## Results

To explore the effects of second hand tobacco smoke exposure on the pulmonary compartment we exposed mice to a mixture of sidestream and mainstream smoke at concentrations similar to what an individual would experience through second hand smoke exposure (Teague et al., 1994; Woodruff et al., 2009). Both naïve wild type C57BL/6 and *Arhgef1*^−/−^ mutant mice were exposed to either FA or SHS for 4 weeks after which time the number of pulmonary leukocytes in lung tissue and airspace were enumerated and characterized.

### Lung tissue leukocytes

**Figure 2:**
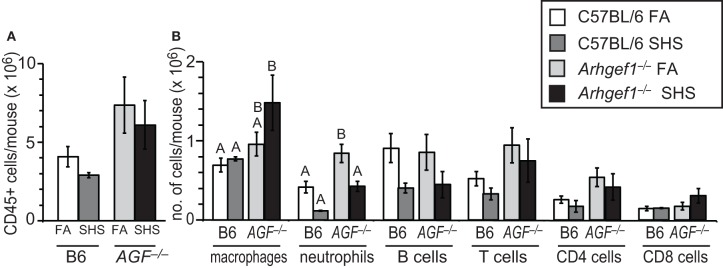
**Leukocyte numbers in lung tissue after 4 weeks of second hand smoke exposure. (A)** Total number of CD45^+^ leukocytes in enzymatically digested and lavaged lung tissue from C57BL/6 (B6) and *Arhgef1*^−/−^ (*AGF*^−/−^) mice exposed to filtered air (FA) or second hand smoke (SHS) for 4 weeks. **(B)** Number of leukocytes within different populations from enzymatically digested and lavaged lung tissue. Shown are the number of macrophages (F4/80^+^), neutrophils (Gr-1^+^), B lymphocytes (B220^+^) and T lymphocytes (CD3^+^), including CD4^+^ and CD8^+^ cells. Three month old C57BL/6 (B6) mice exposed to filtered air (FA) (open bars, *n* = 8), 3 month old C57BL/6 (B6) mice exposed to SHS for 4 weeks prior to harvest (dark gray bars, *n* = 4), 3 month old *Arhgef1*^−/−^ (*AGF*^−/−^) mice exposed to filtered air (FA) (light gray bars, *n* = 7) and 3 month old *Arhgef1*^−/−^ (*AGF*^−/−^) mice exposed to SHS for 4 weeks prior to harvest (black bars, *n* = 4). Data represents mean ± SE. A One-Way ANOVA was performed on leukocyte populations. Statistically significant differences between groups were detected only for macrophages and neutrophils. A *post hoc* Tukey–Kramer HSD *t* test was performed on these groups. Groups not sharing the same letter are significantly different, *P* < 0.05. For the macrophages the B6 FA, B6 SHS, and *AGF*^−/−^ FA groups all share the letter A so none of these groups are significantly different from each other. The *AGF*^−/−^ FA and the *AGF*^−/−^ SHS groups share the letter B so these two groups are not significantly different from each other. The significant difference in macrophages occurs between the *AGF*^−/−^ SHS group (black bar) which only has the letter B designation and the B6 FA and B6 SHS groups (open and light gray bars) which only have the letter A designation. Cells were enumerated and analyzed as described in materials and methods.

To quantify inflammatory cell infiltration in lavaged lung tissue after FA or SHS exposure we utilized enzymatic digestion of lung tissue followed by cell counting and flow cytometry. The results from these analyses reveal that sub-chronic SHS exposure of wild type mice does not lead to a significant change in the total numbers of CD45^+^ leukocytes in lung tissue (Figure 2A). Similarly, the number of total leukocytes in the Arhgef1-deficient lungs did not change following SHS exposure, although there are more pulmonary tissue leukocytes in Arhgef1-deficient lungs compared to the wild type when matched for exposure and as previously reported (Figure 2A and Hartney et al., 2010).

Although SHS exposure did not significantly alter the total number of leukocytes in lung tissue from either wild type or mutant mice, the number of pulmonary tissue macrophages in *Arhgef1*^−/−^ mice was modestly but not significantly increased after 4 weeks of SHS exposure.

In contrast to the increase in macrophages, the neutrophils present in wild type and *Arhgef1*^−/−^ lung tissues decreases after exposure to SHS. This decrease reaches statistical significance for the Arhgef1-deficient cohort (Figure 2B).

### Airspace leukocytes

**Figure 3:**
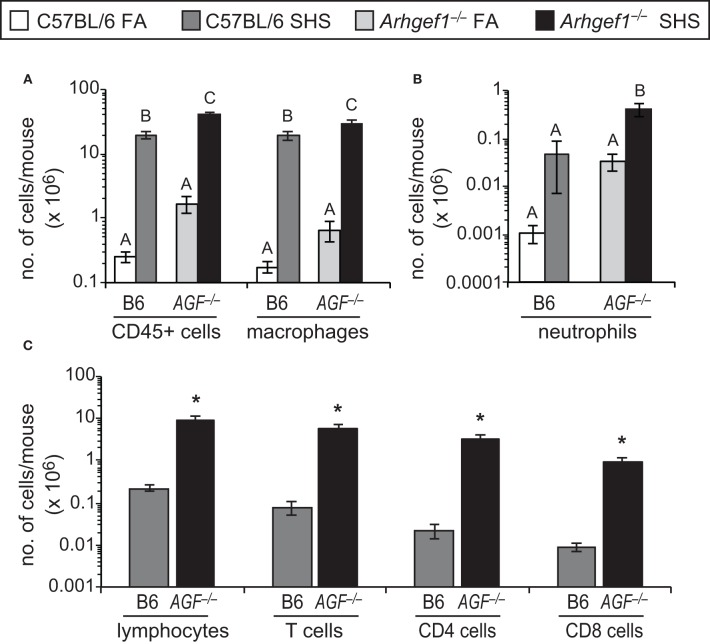
**Second hand smoke exposure for 4 weeks leads to increased numbers of pulmonary leukocytes in the airspace.** Leukocytes were enumerated and characterized from bronchoalveolar lavage (BAL) as defined in materials and methods. **(A)** Total number of CD45^+^ leukocytes and macrophages (F4/80^+^) in BAL from C57BL/6 (B6) and *Arhgef1*^−/−^ (*AGF*^−/−^) mice exposed to filtered air (FA) or second hand smoke (SHS). **(B)** Enumeration of neutrophils (Gr-1^+^) from C57BL/6 (B6) and *Arhgef1*^−/−^ (*AGF*^−/−^) samples as in panel **(A)**. **(C)** Quantitation of lymphocytes, T cells (CD3^+^), CD4 and CD8 cells. Three month old C57BL/6 (B6) mice exposed to FA (open bars, *n* = 8), 3 month old C57BL/6 (B6) mice exposed to SHS for 4 weeks prior to harvest (dark gray bars, *n* = 4), 3 month old *Arhgef1*^−/−^ (*AGF*^−/−^) mice exposed to FA (light gray bars, *n* = 7) and 3 month old *Arhgef1*^−/−^ (*AGF*^−/−^) mice exposed to SHS for 4 weeks prior to harvest (black bars, *n* = 4). Data represents mean ± SE. A One-Way ANOVA was performed for all leukocyte populations. Statistically significant differences between groups were detected in all populations. A *post hoc* Tukey–Kramer HSD *t* test was performed. Groups not sharing the same letter are significantly different, *P* < 0.05. Due to limited number of cells in FA samples quantitation in **C** was only performed on SHS exposed samples. ^*^P < 0.05 Student two-tailed *t* test compared with identically treated C57BL/6 samples.

Despite the modest effect that SHS exposure has on lung tissue leukocyte numbers, we observed a robust and significant increase in the total number of leukocytes recovered in bronchoalveolar lavage (BAL) from both C57BL/6 and *Arhgef1*^−/−^ animals exposed to SHS (Figure 3A). This increase in leukocytes could be largely accounted for by a significant increase in the alveolar macrophages recovered from the BAL of both wild type and mutant lungs (Figure 3A). Neutrophils (Gr-1^+^ cells) were also increased in the BAL of SHS exposed animals compared to FA controls for both genotypes, although this increase was only significant in *Arhgef1*^−/−^ cohort (Figure 3B). We performed a Two-Way ANOVA on our BAL leukocyte quantitation and failed to detect a significant interaction between genotype (C57BL/6 vs. *Arhgef1*^−/−^) and exposure (FA vs. SHS). Because of the increase in leukocytes recovered from SHS exposed animals, we were able to further identify the lymphocyte populations present in these samples (Figure 3C). These findings demonstrate that SHS exposed *Arhgef1*^−/−^ mice harbor significantly more T lymphocytes compared to C57BL/6 mice and is true for both CD4 and CD8 T cell populations (Figure 3C).

### Lung architecture

**Figure 4:**
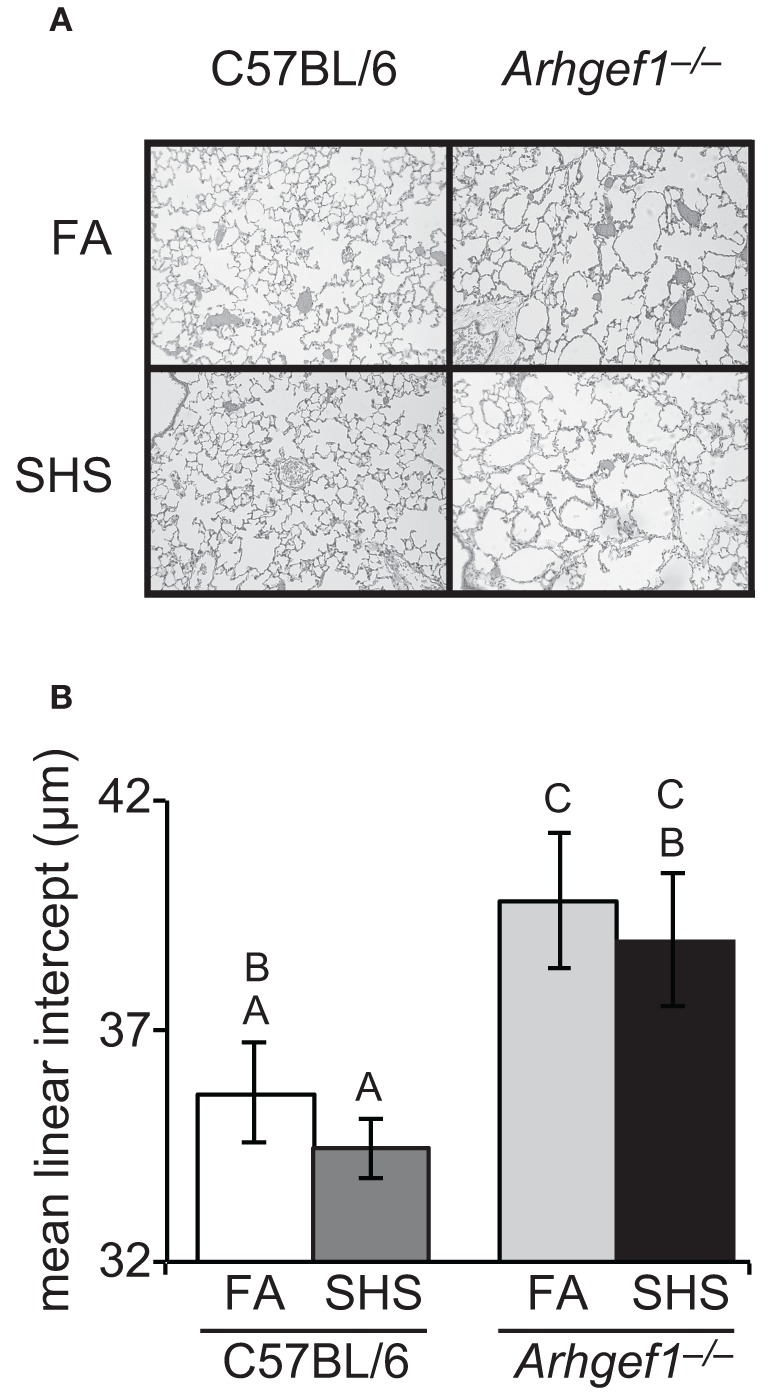
**Short term exposure to second hand cigarette smoke does not lead to airspace enlargement.** Airspace structure was quantitated by mean linear intercept (μm) of alveolar septae as measured on inflated, hematoxylin and eosin-stained lung sections as described in materials and methods. **(A)** Representative lung tissue sections from C57BL6 animals (left column) or *Arhgef1*^−/−^ animals (right column) exposed to either filtered air (FA, top row) or second hand smoke (SHS, bottom row). **(B)** Mean linear intercept (μ m) of 3 month old C57BL/6 mice exposed to filtered air (FA) (open bar, *n* = 7), 3 month old C57BL/6 mice exposed to second hand smoke (SHS) for 4 weeks prior to harvest (dark gray bar, *n* = 8), 3 month old *Arhgef1*^−/−^ mice exposed to FA (light gray bar, *n* = 9) and 3 month old *Arhgef1*^−/−^ mice exposed to SHS for 4 weeks prior to harvest (black bar, *n* = 7). A One-Way ANOVA was performed and significant differences were detected between groups. A *post hoc* Tukey–Kramer HSD *t* test was performed. Groups not sharing the same letter are significantly different, *P* < 0.05.

We next evaluated if SHS exposure and the resulting elevated number of airspace leukocytes altered lung architecture. To accomplish this, we inflated and fixed the lungs of a subset of animals and measured airspace structure as determined by mean linear intercept and described in materials and methods. Representative micrographs from each experimental group are shown in Figure 4A. These data demonstrated that sub-chronic exposure to SHS is not sufficient to alter the airspace structure in the lungs of C57BL/6 mice (Figure 4B). When matched for exposure the *Arhgef1*^−/−^ lungs exhibit significant airspace enlargement compared to the C57BL/6 lungs consistent with our previous report (Figure 4B and Hartney et al., 2010). Although the Arhgef1-deficient mice exhibited an exaggerated inflammatory response, this increased response was not sufficient to induce an increase in airspace enlargement beyond what is already present in the naïve *Arhgef1*^−/−^ animals (Figure 4B).

### Respiratory mechanics

**Figure 5:**
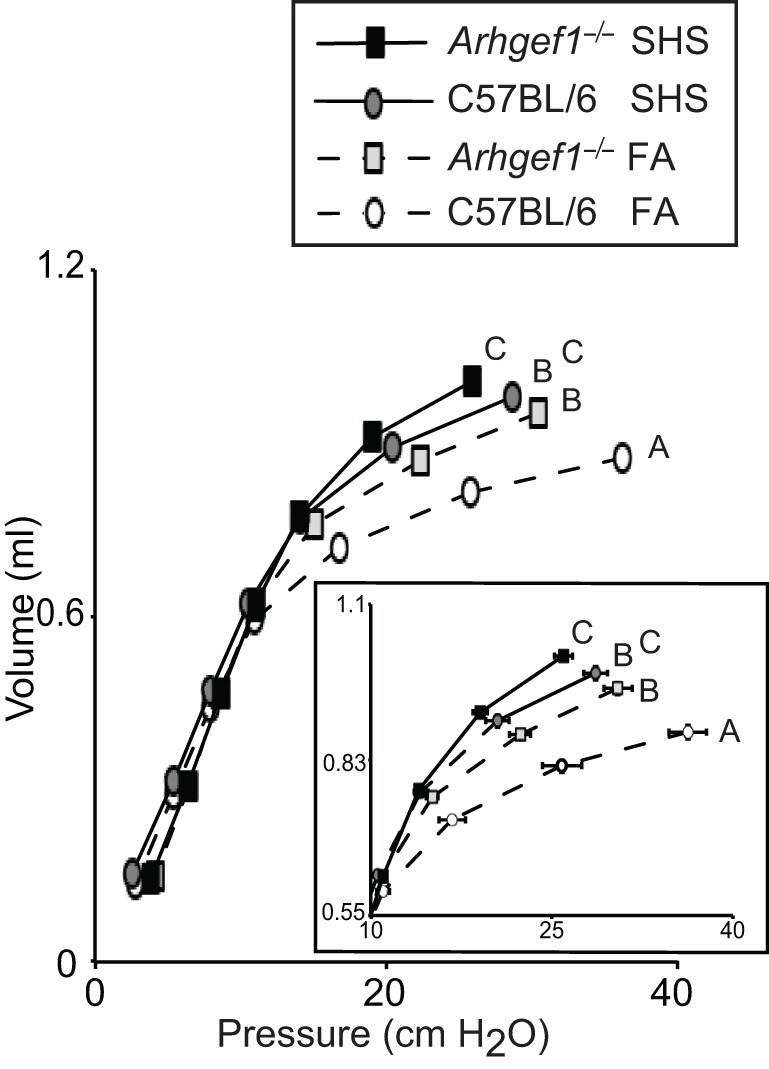
**Lung respiratory mechanics of C57BL/6 and *Arhgef1*^−/−^ mice after 4 weeks of exposure to filtered air (FA) or second hand smoke (SHS).** Measurement of quasi-static pressure-volume loops were performed on 3 month old C57BL/6 mice exposed to FA (open circles, dashed line, *n* = 8), 3 month old *Arhgef1*^−/−^ mice exposed to FA (light gray boxes, dashed line, *n* = 12), 3 month old C57BL/6 mice exposed to SHS for 4 week prior to harvest (dark gray circles, solid line, *n* = 7) and *Arhgef1*^−/−^ mice exposed to SHS for 4 weeks prior to harvest (black boxes, solid line, *n* = 13). Insert shows expanded view of the upper right portion of pressure-volume loop. Data represents mean ± SE. A One-Way ANOVA was performed and significant differences were detected between groups. A *post hoc* Tukey–Kramer HSD *t* test was performed. Groups not sharing the same letter are significantly different, *P* < 0.05.

Prior to leukocyte enumeration and characterization or lung fixation, we assessed lung mechanics in all mice exposed to FA or SHS using a small animal ventilator. Specifically, lung function was assessed by measuring pressure-volume loops (Figure 5). As previously shown for naïve unchallenged mice (Hartney et al., 2010), *Arhgef1*^−/−^ mice exposed to filtered air have significantly decreased lung elastance compared to similarly treated wild type mice. Furthermore, a 4 week exposure to SHS significantly decreased lung elastance in both wild type and mutant animals (Figure 5). Results of a Two-Way ANOVA on these data reveal no interaction between genotype (C57BL/6 vs. *Arhgef1*^−/−^) and exposure (FA vs. SHS). Therefore, we conclude that the pathways leading to decreased lung elastance in the *Arhgef1*^−/−^ mice are independent of the pathways leading to decreased lung elastance after SHS exposure. The results from these experiments demonstrate that sub-chronic SHS exposure is sufficient to significantly decrease lung elastance in both mouse strains examined (Figure 5).

## Discussion

This study documents that 4 week sub-chronic exposure to second hand cigarette smoke does not lead to measureable leukocyte infiltration within lung tissue but does result in airspace inflammation and decreased lung elastance. The inability of sub-chronic smoke exposure to promote lung tissue inflammation or changes in airspace structure is consistent with previous reports (D'Hulst et al., 2005; Rinaldi et al., 2012) and was observed in both C57BL/6 and *Arhgef1*^−/−^ animals. In contrast to the lack of change in lung tissue inflammation and architecture, SHS exposure elicits a robust increase in all leukocyte populations recovered from BAL as previously reported (Woodruff et al., 2009). Within the SHS exposure cohort, all leukocyte populations examined were increased in *Arhgef1*^−/−^ airspace compared to C57BL/6. Characterization of lymphocyte subsets recovered from the BAL of SHS exposed animals also reveal a statistically significant increase in the *Arhgef1*^−/−^ samples compared to identically treated C57BL/6 samples. Together these data demonstrate that even sub-chronic (4 week) exposure to SHS is sufficient to induce significant increases in the number of airspace leukocytes present in either healthy or mutant lungs.

Examination of lung structure in C57BL/6 SHS exposed animals indicates that no morphological changes in lung structure have occurred. This result was not unexpected as it has been previously reported for this mouse strain that at least 3 months of tobacco smoke exposure are required to induce structural changes as measured by an increase in mean linear intercept (Bartalesi et al., 2005). We did hypothesize that the presence of pre-existing inflammation and airspace enlargement in the naïve Arhgef1-deficient mice would decrease the duration of SHS exposure required to induce further pathological changes. However, our current 4 week protocol failed to induce any increase in airspace enlargement in the Arhgef1-deficient animal beyond what is already present in the naïve animals, despite the exaggerated inflammatory response of the *Arhgef1*^−/−^ mice to SHS exposure.

Significant decreases in lung elastance are evident in the C57BL/6 and *Arhgef1*^−/−^ animals when exposed to SHS for a relative short duration (4 weeks). Initially we were surprised to observe a change in lung mechanics with a relative short smoke exposure protocol. A previous report failed to observe any changes in the lung mechanics of C57BL/6 mice exposed to smoke for 6 months (Guerassimov et al., 2004). However close examination of the methods employed to measure lung mechanics reveal an importance difference that may account, at least in part, for the discrepancy between our results and their study. In the previous report the investigators performed a primewave perturbation across a range of positive end expiratory pressures (PEEP) from 3 to 9 cm H_2_O in order to generate a P-V loop (Guerassimov et al., 2004). In our study we generated a pressure-volume loop where lung mechanics are measured over a range of pressures from 2 ~ 30 cm H_2_O. Examination of our own pressure and volume measurements between 2 and 10 cm H_2_O portion of the loop reveal no discernible difference in the SHS exposure groups. We did perform the primewave perturbation at a PEEP of 3 cm H_2_O and consistent with their results do not detect any significant changes in lung elastance in the SHS exposed animals (data not shown). A recent review included both of these measurements performed in the pallid mouse strains (see Figure 4 in Wright et al., 2008). The primewave perturbation yields a modest but significant shift in lung mechanics of the pallid strain (Figure 4A in Wright et al., 2008) while the pressure-volume loop inflating the lungs up to pressures around 30 cm H_2_O demonstrate dramatic differences between the healthy and diseased (pallid) lungs (Figure 4B in Wright et al., 2008). Together these data suggest that a P-V loop which inflates the lungs up to higher pressures (~30 cm H_2_O) may be more sensitive to detecting modest changes in lung elastance than measurement performed at lower pressures.

In addition to the differences in methods of measurements it is also worth noting the difference in duration of smoke exposure protocols between the studies, 4 weeks versus 6 months (Guerassimov et al., 2004). Aside from the increased duration of smoke exposure another parameter to consider is the age of the mice at the time of assessment. Our studies performing lung mechanics measurements find a progressive decrease in lung elastance of C57BL/6 mice from 3 months of age to 1 year of age, similar to reports by other investigators (Huang et al., 2007). Comparison of naïve *Arhgef1*^−/−^ mice and C57BL/6 mice across these ages reveal the most pronounced differences between strains occur at 6 months of age (Hartney et al., 2010).

Comparing the lung mechanics and airspace architecture between all four groups suggests that changes in murine lung mechanics can occur in the presence or absence of changes in lung architecture. Note the SHS exposed C57BL/6 mice have lung elastance values lower than the naïve Arhgef1-deficient mice despite the lack of alteration in airspace structure, as measured by mean linear intercept (Figures 4 and 5). The lack of a direct correlation between lung mechanics and airspace structure has been noted by several investigators examining the effects of cigarette smoke exposure in mouse models (Guerassimov et al., 2004; Foronjy et al., 2005; Rinaldi et al., 2012). Based on these differences it has been proposed that separate pathways are involved in the development of histological alterations in lung architecture versus physiological changes in lung mechanics (Foronjy et al., 2005). Our study provides another instance of these two pulmonary phenotypes occurring independently and supports their proposed hypothesis.

We have previously described a signaling pathway that operates in pulmonary myeloid cells that leads to the production of pro-inflammatory mediators and is normally inhibited by Arhgef1 (Hartney et al., 2011). To address whether this same Arhgef1-regulated pathway contributes to cigarette smoke-induced inflammation we compared the responses of *Arhgef1*^−/−^ and wild type mice to SHS exposure. Using a Two-Way ANOVA, no interaction is found between genotype and response to cigarette smoke exposure in any of our data sets. Thus, we conclude that these pathways, Arhgef1 and cigarette smoke exposure induced responses appear to occur independently of each other.

In conclusion the data presented here demonstrate that sub-chronic SHS exposure is sufficient to induce a significant increase in airspace leukocytes and decrease in lung elastance in both healthy animals and a mutant mouse strain with pre-existing pulmonary inflammation and pathology. This change in lung mechanics appears to occur as a result of processes that can be independent of changes in airspace structure. Further examination of the pathways responsible for SHS induced changes in lung mechanics may identify novel targets for restoring or retaining lung elastance in human subjects exposed to second hand smoke.

### Conflict of interest statement

The authors declare that the research was conducted in the absence of any commercial or financial relationships that could be construed as a potential conflict of interest.

## References

[B1] Barnoya J. Glantz S. A. (2005). Cardiovascular effects of secondhand smoke: nearly as large as smoking. Circulation 111, 2684–2698 10.1161/CIRCULATIONAHA.104.492215 15911719

[B2] Bartalesi B. Cavarra E. Fineschi S. Lucattelli M. Lunghi B. Martorana P. A. Lungarella G. (2005). Different lung responses to cigarette smoke in two strains of mice sensitive to oxidants. Eur. Respir. J. 25, 15–22 10.1183/09031936.04.00067204 15640318

[B3] Centers for Disease Control. (1986). 1986 Surgeon general's report: the health consequences of involuntary smoking. MMWR Morb. Mortal. Wkly. Rep. 35, 769–770 3097495

[B4] D'Hulst A. I. Vermaelen K. Y. Brusselle G. G. Joos G. F. Pauwels R. A. (2005). Time course of cigarette smoke-induced pulmonary inflammation in mice. Eur. Respir. J. 26, 204–213 10.1183/09031936.05.00095204 16055867

[B5] Eisner M. D. Klein J. Hammond S. K. Koren G. Lactao G. Iribarren C. (2005). Directly measured second hand smoke exposure and asthma health outcomes. Thorax 60, 814–821 10.1136/thx.2004.037283 16192366 PMC1747192

[B6] Foronjy R. F. Mercer B. A. Maxfield M. W. Powell C. A. D'Armiento J. Okada Y. (2005). Structural emphysema does not correlate with lung compliance: lessons from the mouse smoking model. Exp. Lung Res. 31, 547–562 10.1080/019021490951522 16019987

[B7] Francis S. A. Shen X. Young J. B. Kaul P. Lerner D. J. (2006). Rho GEF Lsc is required for normal polarization, migration, and adhesion of formyl-peptide-stimulated neutrophils. Blood 107, 1627–1635 10.1182/blood-2005-03-1164 16263795 PMC1895409

[B8] Girkontaite I. Missy K. Sakk V. Harenberg A. Tedford K. Potzel T. Pfeffer K. Fischer K. D. (2001). Lsc is required for marginal zone B cells, regulation of lymphocyte motility and immune responses. Nat. Immunol. 2, 855–862 10.1038/ni0901-855 11526402

[B9] Guerassimov A. Hoshino Y. Takubo Y. Turcotte A. Yamamoto M. Ghezzo H. Triantafillopoulos A. Whittaker K. Hoidal J. R. Cosio M. G. (2004). The development of emphysema in cigarette smoke-exposed mice is strain dependent. Am. J. Respir. Crit. Care Med. 170, 974–980 10.1164/rccm.200309-1270OC 15282203

[B10] Hartney J. M. Brown J. Chu H. W. Chang L. Y. Pelanda R. Torres R. M. (2010). Arhgef1 regulates alpha5beta1 integrin-mediated matrix metalloproteinase expression and is required for homeostatic lung immunity. Am. J. Pathol. 176, 1157–1168 10.2353/ajpath.2010.090200 20093499 PMC2832139

[B11] Hartney J. M. Gustafson C. E. Bowler R. P. Pelanda R. Torres R. M. (2011). Thromboxane receptor signaling is required for fibronectin-induced matrix metalloproteinase 9 production by human and murine macrophages and is attenuated by the arhgef1 molecule. J. Biol. Chem. 286, 44521–44531 10.1074/jbc.M111.282772 22086927 PMC3247948

[B12] Hautamaki R. D. Kobayashi D. K. Senior R. M. Shapiro S. D. (1997). Requirement for macrophage elastase for cigarette smoke-induced emphysema in mice. Science 277, 2002–2004 10.1126/science.277.5334.2002 9302297

[B13] Hu J. Strauch P. Rubtsov A. Donovan E. E. Pelanda R. Torres R. M. (2008). Lsc activity is controlled by oligomerization and regulates integrin adhesion. Mol. Immunol. 45, 1825–1836 10.1016/j.molimm.2007.11.002 18157933 PMC2315659

[B14] Huang K. Rabold R. Schofield B. Mitzner W. Tankersley C. G. (2007). Age-dependent changes of airway and lung parenchyma in C57BL/6J mice. J. Appl. Physiol. 102, 200–206 10.1152/japplphysiol.00400.2006 16946023

[B15] Lovgren A. K. Jania L. A. Hartney J. M. Parsons K. K. Audoly L. P. Fitzgerald G. A. Tilley S. L. Koller B. H. (2006). COX-2-derived prostacyclin protects against bleomycin-induced pulmonary fibrosis. Am. J. Physiol. Lung Cell. Mol. Physiol. 291, L144–L156 10.1152/ajplung.00492.2005 16473862

[B16] Ma B. Kang M. J. Lee C. G. Chapoval S. Liu W. Chen Q. Coyle A. J. Lora J. M. Picarella D. Homer R. J. Elias J. A. (2005). Role of CCR5 in IFN-gamma-induced and cigarette smoke-induced emphysema. J. Clin. Invest. 115, 3460–3472 10.1172/JCI24858 16284650 PMC1280966

[B17] Martin R. J. Wexler R. B. Day B. J. Harbeck R. J. Pinkerton K. E. Chu H. W. (2006). Interaction between cigarette smoke and mycoplasma infection: a murine model. COPD 3, 3–8 17175659 10.1080/15412550500493162

[B18] Oberg M. Jaakkola M. S. Woodward A. Peruga A. Pruss-Ustun A. (2011). Worldwide burden of disease from exposure to second-hand smoke: a retrospective analysis of data from 192 countries. Lancet 377, 139–146 10.1016/S0140-6736(10)61388-8 21112082

[B19] Rinaldi M. Maes K. De Vleeschauwer S. Thomas D. Verbeken E. K. Decramer M. Janssens W. Gayan-Ramirez G. N. (2012). Long-term nose-only cigarette smoke exposure induces emphysema and mild skeletal muscle dysfunction in mice. Dis. Model Mech. 3, 333–341 10.1242/dmm.008508 22279084 PMC3339827

[B20] Rubtsov A. Strauch P. Digiacomo A. Hu J. Pelanda R. Torres R. M. (2005). Lsc regulates marginal-zone B cell migration and adhesion and is required for the IgM T-dependent antibody response. Immunity 23, 527–538 10.1016/j.immuni.2005.09.018 16286020

[B21] Subramaniam M. Bausch C. Twomey A. Andreeva S. Yoder B. A. Chang L. Crapo J. D. Pierce R. A. Cuttitta F. Sunday M. E. (2007). Bombesin-like peptides modulate alveolarization and angiogenesis in bronchopulmonary dysplasia. Am. J. Respir. Crit. Care Med. 176, 902–912 10.1164/rccm.200611-1734OC 17585105 PMC2048672

[B22] Teague S. V. Pinkerton K. E. Goldsmith M. Gerbremicheal A. Chang S. Jenkins R. A. Moneyhun J. H. (1994). Sidestream cigarette smoke generation and exposure system for environmental tobacco smoke studies. Inhal. Toxicol. 6, 79–93

[B23] Tollefson A. K. Oberley-Deegan R. E. Butterfield K. T. Nicks M. E. Weaver M. R. Remigio L. K. Decsesznak J. Chu H. W. Bratton D. L. Riches D. W. Bowler R. P. (2010). Endogenous enzymes (NOX and ECSOD) regulate smoke-induced oxidative stress. Free Radic. Biol. Med. 49, 1937–1946 10.1016/j.freeradbiomed.2010.09.022 20887783 PMC3780970

[B24] Tschanz S. A. Burri P. H. (2002). A new approach to detect structural differences in lung parenchyma using digital image analysis. Exp. Lung Res. 28, 457–471 10.1080/01902140290096719 12217212

[B25] Woodruff P. G. Ellwanger A. Solon M. Cambier C. J. Pinkerton K. E. Koth L. L. (2009). Alveolar macrophage recruitment and activation by chronic second hand smoke exposure in mice. COPD 6, 86–94 10.1080/15412550902751738 19378221 PMC2873864

[B26] Wright J. L. Cosio M. Churg A. (2008). Animal models of chronic obstructive pulmonary disease. Am. J. Physiol. Lung Cell. Mol. Physiol. 295, L1–L15 10.1152/ajplung.90200.2008 18456796 PMC2494776

